# Quality of life and kidney function in CKD patients: a longitudinal study

**DOI:** 10.1093/ckj/sfae429

**Published:** 2025-03-24

**Authors:** Graziella D'Arrigo, Carmela Marino, Patrizia Pizzini, Graziella Caridi, Francesco Marino, Giovanna Parlongo, Annalisa Pitino, Mercedes Gori, Giovanni Tripepi, Francesca Mallamaci, Carmine Zoccali

**Affiliations:** National Research Council of ITALY (CNR) Institute of Clinical Physiology in Reggio Calabria, Italy; National Research Council of ITALY (CNR) Institute of Clinical Physiology in Reggio Calabria, Italy; National Research Council of ITALY (CNR) Institute of Clinical Physiology in Reggio Calabria, Italy; Nephrology and Renal Transplantation Unit, Grande Ospedale Metropolitano, Reggio Calabria, Italy; Nephrology and Renal Transplantation Unit, Grande Ospedale Metropolitano, Reggio Calabria, Italy; Nephrology and Renal Transplantation Unit, Grande Ospedale Metropolitano, Reggio Calabria, Italy; National Research Council of ITALY (CNR) Institute of Clinical Physiology in Rome; National Research Council of ITALY (CNR) Institute of Clinical Physiology in Rome; National Research Council of ITALY (CNR) Institute of Clinical Physiology in Reggio Calabria, Italy; National Research Council of ITALY (CNR) Institute of Clinical Physiology in Reggio Calabria, Italy; Nephrology and Renal Transplantation Unit, Grande Ospedale Metropolitano, Reggio Calabria, Italy; Renal Research Institute, NY, USA; Institute of Molecular Biology and Genetics (Biogem), Ariano Irpino, Italy; Associazione Ipertensione Nefrologia Trapianto (IPNET), c/o Nefrologia, Grande Ospedale Metropolitano, Reggio Calabria, Italy

**Keywords:** chronic kidney disease, glomerular filtration rate, longitudinal study, mixed linear models, quality of life

## Abstract

**Background:**

In chronic kidney disease (CKD), a declining glomerular filtration rate (GFR) leads to physiological and psychosocial burdens that impair quality of life (QoL). However, this relationship has been mainly investigated in cross-sectional studies and a limited number of longitudinal studies that reported contrasting results.

**Objective and methods:**

This longitudinal study included 582 patients with Stages 2–5 CKD. QoL was assessed using the Kidney Disease Quality of Life (KDQOL™) instrument at baseline and annually for 3 years. GFR was estimated using the MDRD equation, the equation recommended by guidelines that were contemporary to the study cohort. We analysed the relationship between repeated measures of QoL and GFR using unadjusted and adjusted mixed linear models (MLMs).

**Results:**

The cohort had a mean age of 61 ± 12 years, with 60% males and 33% diabetics. Baseline eGFR was 36±13 ml/min/1.73 m². Physical and mental component summary scores of QoL were 43.5 and 45.1, respectively. In MLM analyses adjusted for potential confounders, a 10 ml/min/1.73 m² decrease in eGFR was significantly associated with reductions in physical (*β *=* *−0.60, *P *= .016) and mental (*β *=* *−0.52, *P *= .045) component summary scores over the follow-up period. The physical functioning and role limitation physical health subcomponents of QoL were primarily responsible for these associations.

**Conclusions:**

This longitudinal study shows that declining kidney function is associated with deteriorating QoL in CKD patients independently of other factors. These findings support the current KDIGO recommendation that regular monitoring of QoL should be incorporated into clinical practice to improve patient outcomes.

KEY LEARNING POINTS **What was known**:

- Previous studies indicated that chronic kidney disease (CKD) adversely affects the quality of life (QoL) due to declining kidney function, but most were cross-sectional, lacking insights into longitudinal changes.
- The relationship between glomerular filtration rate (GFR) decline and QoL deterioration was poorly understood.
- There was a need for comprehensive studies to explore how CKD progression affects QoL longitudinally, accounting for socio-economic and comorbid factors.

**This study adds**:

- This study establishes a clear link between declining GFR and reduced QoL over 3 years, highlighting physical and mental health effects.
- It demonstrates that physical functioning and role limitation physical health are the primary QoL domains affected by kidney function decline.
- The findings support integrating regular QoL assessments into CKD management to address medical and psychosocial patient needs.

**Potential impact**:

- These insights advocate for routine QoL monitoring in CKD care, potentially improving patient-centred treatment strategies.
- Policymakers may consider incorporating QoL metrics into clinical guidelines to enhance CKD management and patient outcomes.
- The study underscores the importance of addressing socio-economic and comorbid factors in CKD treatment plans to improve overall QoL.

## INTRODUCTION

Chronic kidney disease (CKD) is a global health concern [1], with its prevalence and impact on patient well-being drawing increasing attention from the medical community [2]. CKD is characterized by a progressive decline in glomerular filtration rate (GFR), a critical marker of kidney function. As CKD progresses, this decline in GFR leads to a cascade of physiological and psychosocial burdens that significantly impair the quality of life (QoL) of affected individuals [3]. Health-related quality of life (HRQoL) is a multidimensional concept that encompasses physical, psychological, and social domains of health, all of which can be adversely affected by CKD. The impact of CKD on HRQoL is profound, as patients often experience symptoms such as fatigue, pain, and emotional distress, which can severely limit daily activities and overall well-being. For patients, the essence of their coping with disease lies in the QoL, transcending mere survival to embrace well-being and fulfilment. Moreover, the treatment modalities for CKD, including dialysis and transplantation, introduce additional challenges that further complicate the QoL of patients.

The interplay between QoL and GFR has been mainly investigated in cross-sectional studies, which provide only a snapshot of the relationship between QoL and GFR at a single point in time [4–6]. While valuable, cross-sectional analyses are inherently inadequate to capture this association's evolution and potential bidirectional nature. To address this gap, our study applies a longitudinal design, wherein repeated measures of QoL and GFR and pertinent potential confounders are collected over time from a large, well-characterized cohort of CKD patients. This is essential because QoL can be influenced by kidney disease progression and the concomitant worsening of other medical conditions.

In this study, we aim to provide a holistic understanding of the QoL burden in CKD through repeated measures and statistical modelling, which can better explain the temporal dynamics of the QoL and GFR relationship.

## MATERIALS AND METHODS

This is a longitudinal study investigating the relationship between QoL and kidney function over a 3-year follow-up. The study protocol conformed to our institution's ethical guidelines, and informed consent was obtained from each participant.

### Patients

The original study cohort included 759 patients [7]. In this analysis, we included 582 patients with Stages 2–5 CKD who completed the QoL questionnaires consecutively recruited from nephrology units in Southern Italy (Calabria, Sardinia, and Sicily regions). All these patients were enrolled in the MAURO study (a cluster randomized controlled trial) in 22 renal clinics to assess the efficacy of a multimodal quality improvement intervention to increase compliance with guideline recommendations for preventing CKD progression and CV complications. Patient enrolment was performed between October 2005 and 2008. The selection criteria and the detailed clinical characteristics of this cohort were described elsewhere [7, 8]. A detailed description of the present study cohort is given in Table 1. The study included six visits at prefixed times over a 3-year follow-up. At enrolment, all patients were in stable clinical condition and none had inter-current infections or acute inflammatory processes. Inclusion criteria were non-acute or rapidly evolving renal diseases, age ranging from 18 to 75 years, non-transplanted and non-pregnant, and not affected by cancer or diseases in the terminal phase.

**Table 1:** Demographic, clinical, biochemical data, and QoL questionnaire of study patients at baseline.

Demographic, clinical, and biochemical factors	Whole population (*n* = 582)
Age, years	61 ± 12
Men, %	60%
BMI, kg/m^2^	28 ± 5
Smokers, %	49%
Diabetics, %	33%
Cardiovascular comorbidities, %	29%
Systolic BP (mmHg)	133 ± 18
Diastolic BP (mmHg)	77 ± 11
Haemoglobin (g/dl)	12.9 ± 1.8
Total cholesterol (mg/dl)	189 ± 44
HDL cholesterol (mg/dl)	51 ± 17
LDL cholesterol (mg/dl)	108 ± 37
Calcium (mg/dl)	9.4 ± 0.7
Phosphate (mg/dl)	3.7 ± 0.8
Albumin (g/dl)	4.2 ± 0.5
CRP (mg/l)	2.2 (1.0–5.2)
eGFR (ml/min/1.73 m^2^)	36 ± 13
**QoL questionnaire**	
Physical functioning	75 (50–90)
Role limitation physical health	75 (0–100)
Role limitation emotional problem	66.7 (0–100)
Energy/fatigue	55 (35–70)
Emotional	60 (44–76)
Social functioning	75 (50–87.5)
Pain	65 (45–90)
General health	40 (25–55)
MCS	45.1 (34.9–53.5)
PCS	43.5 (35.2–50.5)

### Office blood pressure measurements

Office blood pressure (BP) was calculated as the average of two or three measurements at 1‐ to 2‐minute intervals during the morning hours. BP measurements were taken in a sitting position by the attending physician or a nurse with the cuff at heart level using sphygmomanometers, periodically tested and appropriately calibrated.

### Laboratory measurements

Blood sampling was performed at baseline and during the follow-up visits. The estimated glomerular filtration rate (eGFR) was estimated by the MDRD 186 study equation [9], which was the most used formula when this longitudinal study was designed and performed (2005–2008) and the specific formula adopted in the MAURO study [7, 8]. Notably, in patients with CKD, an eGFR <60 ml/min/1.73 m^2^ is equally reliable to the CKD-EPI formula [10].

Serum lipids, glucose, albumin, calcium, phosphate, albumin, high-sensitivity C-reactive protein (CRP), and haemoglobin were measured by standard methods in routine clinical laboratories of participating centres.

### Quality of life (QoL) assessment

QoL was measured using the short form of Kidney Disease Quality of Life (KDQOL^TM^), which is one of the most applied questionnaires for testing QoL in CKD and KF patients [11]. This questionnaire was validated explicitly for Italian CKD patients [12].

This instrument measures eight domains of QoL (physical functioning, role physical health, energy fatigue, pain, role emotional problem, emotional well-being, social function, and general health) and two summary scores (the physical component score and the mental component score), which are calculated by a well-validated algorithm [13]. In all patients (*n* = 582), the KDQOL^TM^ was administered at enrolment and after 1 (*n* = 489 patients), 2 (*n* = 434) and 3 (*n* = 387) years. Patients who died and those who started dialysis treatment were censored at the date of last observation and of kidney transplant.

The data analysis used a socio-economic indicator risk score based on income and educational level [14] to make an allowance for socio-economic factors.

### Statistical analysis

Continuous variables were summarized as mean and standard deviation (normally distributed data) or as median and interquartile range (non-normally distributed data). Categorical data were expressed as percentages. The Pearson product-moment correlation coefficient (*r*) and *P* value assessed the association between two continuous variables considered simultaneously.

The independent correlates of baseline mental component summary (MCS) and physical component summary (PCS) were identified by univariate and multivariate linear regression analyses. All variables significantly related to baseline MCS and PCS at univariate analyses were introduced into multivariate linear regression models having MCS or PCS as dependent variables.

The independent relationship between eGFR over time and repeated measurements of PCS and MCS were analysed by mixed linear models of different complexity. As potential confounders, we considered all baseline variables listed in Table 1, which resulted in being significantly related to PCS (number of visit, age, gender, BMI, smoking, diabetes, background cardiovascular comorbidities, systolic BP, haemoglobin, calcium, phosphate, albumin, and CRP) and MCS (diabetes, background CV comorbidities, systolic BP, haemoglobin, calcium, phosphate) at univariate MLM (*P *< .05) [4]. These variables were jointly introduced into multiple MLMs In such models, we always forced the socio-economic status indicator, and baseline PCS and MCS. Data were expressed as standardized regression coefficients (beta) and *P* values.

All calculations were made using standard statistical packages (IBM SPSS for Windows V.29.0.1.0; STATA 16 for Windows, College Station, TX, USA).

## RESULTS

The source population consisted of 759 Stage 2–5 CKD patients. Five hundred and eighty-two patients compiled the QoL questionnaire (i.e. 77% of the original study cohort), and all these patients were considered for the present analysis. Their mean age was 61 ± 12 years; 60% were males, 33% were diabetics, and 29% had background cardiovascular comorbidities. Estimated GFR was, on average, 36±13 ml/min/1.73 m^2^. The demographic, clinical, QoL, and socio-economic data are reported in Table 1. Patients were being treated following recommendations of contemporary KDOQI CKD guidelines [15], and treatments were maintained unchanged or underwent minor changes over the follow-up.

A comparison between included (*n* = 582) and excluded (*n* = 177) patients showed that patients who could not be included in the study were more frequently diabetics, with higher systolic BP and CRP and lower haemoglobin and total cholesterol than those considered in the analysis (see online Supplementary Data, Supplementary Table).

Overall, 579 QoL and simultaneous eGFR measurements were performed in the 582 patients participating in this study. In detail, 579 patients had paired measurements of these variables at baseline, 464 after 1 year, 409 after 2 years, and 361 after 3 years.

As described in Table 1, physical functioning, role limitation, and physical health were the QoL domains with the highest values in our study population, while energy/fatigue and general health had the lowest values. Overall, mental and physical component summaries were 45.1 and 43.5, respectively, weakly but still significantly interrelated (*r* = 0.20, *P *< .001). At baseline, the PCS was directly related to eGFR (*r* = 0.13, *P *= .002), whereas MCS did not (*r* = −0.002, *P *= .96). The remaining univariate correlates of baseline MCS and PCS are reported in Supplementary Table S2. In multivariate linear regression models, including all univariate correlates of MCS and PCS, male sex (*β* = 0.14), diabetes (*β* = −0.10), and serum calcium (*β* = 0.09) (all *P* ≤ .03) maintained an independent relationship with baseline MCS whereas background CV comorbidities (*β* = −0.19), age (*β* = −0.15), BMI (*β* = −0.14), haemoglobin (*β* = 0.12), smoking (*β* = 0.11), and diabetes (*β* = −0.09) (all *P* ≤ 0.04) had an independent association with PCS.

### Longitudinal analysis: repeated measurements of physical and mental component scores over time and the eGFR

**Figure 1:**
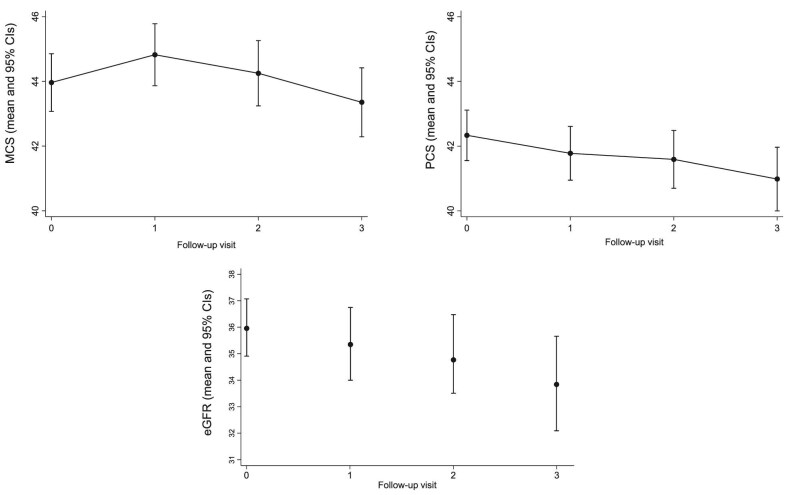
Evolution over time of PCS, MCS, and eGFR. Linear mixed model-derived PCS and MCS data. eGFR data are those observed.

The evolution over time of PCS, MCS, and eGFR is reported in Fig. 1. To study the relationship between QoL and the eGFR over time, we applied mixed linear modelling (MLM). In MLMs of increasing complexity (Table 2), a 10 ml/min/1.73 m^2^ decrease in eGFR over the 3-year follow-up period was associated with a highly significant reduction in the summary measures of QoL, i.e. the PCS and the MCS scores independently of an extensive series of potential confounders (all variables listed in Table 1, see Methods, Statistical Analysis), including the socio-economic status and the corresponding baseline values of PCS and MCS of QoL. The links between repeated PCS and MCS with simultaneous eGFR measurements over time were mainly due to the physical functioning (*P *= .038) and role limitation physical health (*P *= .042) subcomponents of the same scores. Emotional problems (*P *= .374), energy/fatigue (*P *= .145), role emotional (*P *= .494), social functioning (*P *= .275), pain (*P *= .106), and general health (*P *= 0.067) failed to be significantly related to repeated measurement of eGFR.

**Table 2:** Linear mixed model analyses with PCS and MCS scores as dependent variables and repeated measurements of eGFR as key independent variable.

	Model 1^a^	Model 2	Model 3	Model 4
**Dependent variable PCS score**				
eGFR (−10 ml/min/1.73 m^2^)	−1.06 (from −1.48 to −0.63), *P *< .001	−0.64 (from −0.98 to −0.31), *P *< .001	−0.59 (from −1.01 to −0.17), *P *= .005	−0.60 (from −1.10 to −0.11), *P *= .016
	Model 1^b^	Model 2	Model 3	Model 4
**Dependent variable MCS score**				
eGFR (−10 ml/min/1.73 m^2^)	−0.63 (from −1.08 to −0.18), *P *= .007	−0.63 (from −1.01 to −0.25), *P *= .001	−0.50 (from −0.93 to −0.06), *P *= .025	−0.52 (from −1.03 to −0.01), *P *= .045

a Model 1: adjusted for the number of visits.

Model 2: Model 1 + baseline PCS

Model 3: Model 2 + age, gender, BMI, smoking, diabetes, background CV comorbidities, systolic BP, haemoglobin, calcium, phosphate, albumin, and CRP

Model 4: Model 3 + educational levels, socio-economic status

b Model 1: adjusted for the number of visits.

Model 2: Model 1 + baseline MCS

Model 3: Model 2 + diabetes, background CV comorbidities, systolic BP, haemoglobin, calcium, phosphate

Model 4: Model 3 + educational levels, economic status

## DISCUSSION

In this longitudinal study, repeated measurements of physical and mental components of QoL assessed by the short form of the Kidney Disease Quality of Life (KDQOL™) instrument were coherently associated with estimated GFR (eGFR) levels over time.

CKD is a progressive condition characterized by the gradual loss of kidney function over time. It affects millions of individuals worldwide [2] and poses significant challenges to physical health and the overall QoL [3]. The concept of QoL encompasses many factors, including physical health, psychological state, level of independence, social relationships, and personal beliefs. In the context of CKD, QoL is a critical outcome measure that reflects the broader impact of the disease and its treatment on patients’ lives.

The physical health of individuals with CKD is often compromised due to the disease's systemic effects. Symptoms such as fatigue, pain, and sleep disturbances are common and can severely limit daily activities. Additionally, CKD patients frequently experience comorbid conditions such as cardiovascular disease, diabetes, and hypertension, which further exacerbate their physical health challenges. The burden of these symptoms and comorbidities significantly diminishes QoL, an issue noted in a recent, large cohort of non-dialysis-CKD patients across three diverse countries [16]. The psychological impact of CKD is profound [3, 4].

The importance of QoL in CKD management cannot be overstated [3]. Traditional clinical outcomes such as survival rates and biochemical markers, while necessary, do not fully capture the patient's experience of living with CKD. QoL measures provide a more holistic view of the disease's impact and its treatment, guiding healthcare providers in delivering patient-centred care.

To accurately measure HRQoL in CKD patients, the KDQOL™) is the most used instrument on a world scale [11]. KDQOL™ is a self-reported measure that includes a 36-item health survey as the generic core, supplemented with multi-item scales targeted at particular concerns of individuals with kidney disease and on dialysis. The short form (SF) of this instrument, known as the KDQOL-36™, includes the SF-12 as the generic core plus the burden of kidney disease, symptoms/problems of kidney disease, and effects of kidney disease scales from the KDQOL-SF™v1.3. The KDQOL™ instrument has been validated in both the general and CKD populations, ensuring its reliability and relevance across different groups. Specifically, it has been validated in the CKD population in Italy [12], demonstrating its applicability and cultural sensitivity in diverse settings. This validation underscores the instrument's robustness in capturing the unique QoL concerns of CKD patients, making it a valuable tool for both clinical practice and research. Patient related outcome measures such as pain, fatigue, mobility, and other physical symptom capture patient perspectives on health and QoL, crucial for CKD management. They offer comprehensive, patient-centric insights but may be subjective and complex. The KDQOL-36™ is a validated, disease-specific tool for CKD, providing broad health assessments, although it can be lengthy and complex to interpret. Balancing its use with patient burden and cultural adaptation is essential.

CKD presents a complex interplay of physiological decline and psychosocial burden, significantly impacting patients' QoL as the disease progresses. Several cross-sectional studies have documented the association between decreasing GFR and QoL [4–6].

The longitudinal association between CKD progression and QoL deterioration has been investigated in three studies in adults with CKD, but the findings are partly contradictory. Mujais *et al.* [17] found that the cumulative burden of symptoms and complications intensifies as CKD progresses, thereby reducing QoL. Female gender, the presence of diabetes, and a history of cardiovascular comorbidities were the main correlates of reduced QoL. Kilshaw *et al.* [18], in a small series of 41 CKD patients, observed a significant functional deterioration, as detected by the Timed Up and Go test, over the 6 months before death, paralleled by a significant decrease in health-related QoL. In the Australian Diabetes, Obesity, and Lifestyle Study [19], physical but not mental QoL was significantly impaired in CKD and continued to decline with disease progression. Among patients in the Chronic Renal Insufficiency Cohort (CRIC) [20], eGFR decline was only weakly related to changes in patient-reported metrics over time.

Our study longitudinally mapped the trajectories of QoL against GFR decline in a well-characterized cohort of CKD patients. Our findings indicate a commensurate decrease in both the mental and physical components of QoL as kidney function declines. The longitudinal data from our cohort suggest that interventions aimed at slowing kidney function decline could also preserve or improve QoL and vice versa. Differences in race composition (ours is a purely Caucasian cohort, while 42% of patients in CRIC were Blacks) and in comorbidities (diabetes prevalence is 34% in our cohort and 48% in CRIC) may explain the different relationships between the eGFR and QoL metrics in the two studies.

Our findings underscore the need for regular monitoring of QoL as a component of patient-centred care in CKD management. This aligns with the Kidney Disease: Improving Global Outcomes (KDIGO) recommendations, which advocate for integrating patient-reported outcomes into clinical practice to enhance care for CKD patients [21]. We controlled for the potential confounding effect of comorbidities and socio-economic status. This is crucial since these factors have been shown to influence QoL independently of kidney function.

Our study provides novel information on the impact of CKD on specific domains of QoL. In a longitudinal setting, we observed that both physical and mental health domains deteriorate over time. We also observed that traditional risk factors for CKD progression, such as hypertension and diabetes, were associated with lower baseline QoL scores, which is in line with findings from the CRIC cohort [19]. These observations emphasize the importance of effectively managing comorbidities to mitigate their impact on kidney function and QoL.

This study has limitations. First, excluded patients were more frequently diabetics, had higher systolic BP and CRP levels, and lower haemoglobin and total cholesterol levels compared to those included. We dealt with the potential selection bias by properly adjusting the analysis for the significantly different variables among the two groups. However, the possibility of residual confounding cannot be excluded *a priori*. Second, the study cohort was recruited from specific regions in Southern Italy, which may limit the generalizability of the findings to other populations with different demographic and clinical characteristics. Third, the study used the MDRD equation to estimate GFR, which was the standard at the time of our research but has since been replaced by more accurate methods such as the CKD-EPI equation. However, the MDRD and the CKD-EPI equations perform equally well in patients with established CKD [21], as in our study. Fourth, although the study adjusted for several potential confounders, there may still be unmeasured variables that could influence the relationship between kidney function and QoL, such as lifestyle factors or other comorbid conditions.

In conclusion, this study found that a decline in kidney function was significantly associated with reductions in both physical and MCS scores of QoL over the 3-year follow-up period.

The physical functioning and role limitation physical health subcomponents were primarily responsible for the observed associations between eGFR decline and QoL. Other domains, such as energy/fatigue and general health, did not significantly correlate with eGFR. The study utilized a longitudinal design with repeated measures of QoL and GFR over 3 years, allowing for a more nuanced understanding of how changes in kidney function over time influence QoL. The findings highlight the importance of integrated CKD management strategies that preserve kidney function and enhance QoL. Regular monitoring of QoL should be incorporated into clinical practice to improve patient outcomes.

Overall, the study underscores the interconnectedness of kidney function and QoL in CKD patients and suggests that interventions to slow kidney function decline could also help maintain or improve QoL.

## Supplementary Material

sfae429_Supplemental_File

## Data Availability

This study's data will be made available to interested investigators for 6 months after its publication.
