# Supplementary material for: Quality of life and kidney function in CKD patients: a longitudinal study
Source: Clin Kidney J. 2025 Mar 24;18(4):sfae429. doi: 10.1093/ckj/sfae429 (PMC11976526; doi:10.1093/ckj/sfae429)
Supplement: sfae429_Supplemental_File [file sfae429_supplemental_file.docx]

**Supplementary Table S1a** - Comparison between the study cohort and patients not included into the study for demographic data

|  | **Study Cohort**  **(n=582)** | **Patients not included in to the study (n=177)** | **p-value** |
| --- | --- | --- | --- |
| Age, yr | 61±12 | 62±9 | 0.22 |
| Men, % | 60% | 61% | 0.70 |
| **Schooling** |  |  |  |
| Illiterate, % | 7.8% | 5.3% | 0.10 |
| reading and writing, % | 10.8% | 13.3% |  |
| primary school, % | 41.6% | 32.1% |  |
| middle school, % | 23.5% | 25.8% |  |
| high school, % | 13.9% | 18.1% |  |
| degree or over, % | 2.4% | 5.3% |  |
| **Marital** **status** |  |  |  |
| Unmarried, % | 9.5% | 8.7% | 0.73 |
| Married, % | 76.2% | 78.9% |  |
| Widowed, % | 12.5% | 9.9% |  |
| Divorced/separated, % | 1.8% | 2.4% |  |
| Working situation, % | 28.3% | 29.5% | 0.78 |
| Retirement, % | 98.3% | 96.0% | 0.24 |
| Housing property, % | 88.6% | 90.6% | 0.45 |
| **Cohausing** |  |  |  |
| Alone, % | 10.7% | 9.2% | 0.32 |
| with the spouse or the in-home nurse only, % | 29.8% | 36.0% |  |
| with relatives and or other cohabitants, % | 59.5% | 54.8% |  |
| **Number of cars** |  |  |  |
| None, % | 11.8% | 12.4% | 0.38 |
| One, % | 65.2% | 58.0% |  |
| Two, % | 18.0% | 23.0% |  |
| Three or more, % | 5.0% | 6.6% |  |
| **Income status** |  |  |  |
| No income, % | 14.4% | 20.0% | 0.43 |
| Income without car, % | 10.0% | 10.3% |  |
| Income with less than one car per capita, % | 65.0% | 59.4% |  |
| Income with one or more cars per capita, % | 10.6% | 10.3% |  |

**Supplementary Table S1b** - Comparison between the study cohort and patients not included in the study for clinical and biochemical data

|  | **Study Cohort**  **(n=582)** | **Patients not included in to the study (n=177)** | **p-value** |
| --- | --- | --- | --- |
| BMI, Kg/m2 | 28±5 | 28±4 | 0.64 |
| Smokers, % | 49% | 52% | 0.48 |
| **Diabetics, %** | **33%** | **41%** | **0.04** |
| Background cardiovascular comorbidities (%) | 29% | 30% | 0.67 |
| **Systolic BP (mmHg)** | **133±18** | **136±18** | **0.022** |
| Diastolic BP (mmHg) | 77±11 | 78±10 | 0.16 |
| **Hemoglobin (g/dL)** | **12.9±1.8** | **12.5±1.8** | **0.013** |
| **Total cholesterol (mg/dL)** | **189±44** | **180±44** | **0.022** |
| HDL cholesterol (mg/dL) | 51±17 | 48±15 | 0.11 |
| LDL cholesterol (mg/dL) | 108±37 | 102±34 | 0.06 |
| Calcium (mg/dL) | 9.4±0.7 | 9.4±0.5 | 0.38 |
| Phosphate (mg/dL) | 3.7±0.8 | 3.7±0.8 | 0.82 |
| Albumin (g/dL) | 4.2±0.5 | 4.2±0.6 | 0.99 |
| **CRP (mg/L)** | **2.2 (1.0-5.2)** | **2.6 (1.24-6.81)** | **0.04** |
| eGFR(mil/min/1.73m2) | 36±13 | 35±14 | 0.46 |

**Supplementary Table S2** Correlation matrix of baseline MCS and PCS values

|  | MCS | PCS |
| --- | --- | --- |
|  | rho (p-value) | |
| Age | 0.05 (p=0.25) | **-0.21 (p<0.001)** |
| Sex | **0.17 (p<0.001)** | **0.11 (p=0.01)** |
| BMI | -0.01 (p=0.89) | **-0.21 (p<0.001)** |
| Smoking | 0.06 (p=0.15) | **0.11 (p=0.01)** |
| Diabetes | **-0.1 (p=0.02)** | **-0.22 (p<0.001)** |
| Cardiovascular comorbidities | -0.05 (p=0.21) | **-0.25 (p<0.001)** |
| Systolic BP | -0.05 (p=0.21) | -0.06 (p=0.13) |
| Diastolic BP | -0.05 (p=0.29) | **0.09 (p=0.04)** |
| Haemoglobin | **0.17 (p<0.001)** | **0.22 (p<0.001)** |
| Total cholesterol | -0.02 (p=0.59) | 0.06 (p=0.16) |
| HDL cholesterol | -0.02 (p=0.59) | **0.10 (p=0.02)** |
| LDL cholesterol | -0.04 (p=0.37) | 0.08 (p=0.07) |
| Calcium | **0.11 (p=0.01)** | 0.06 (p=0.16) |
| Phosphate | -0.06 (p=0.15) | **-0.15 (p<0.001)** |
| Albumin | **0.11 (p=0.02)** | **0.11 (p=0.02)** |
| CRP | **-0.08 (p=0.05)** | **-0.2 (p<0.001)** |
| eGFR | 0.008 (p=0.85) | **0.12 (0.003)** |
| PCS | **0.20 (p<0.001)** |  |
